# Associations between blood pressure control and clinical events suggestive of nutrition care documented in electronic health records of patients with hypertension

**DOI:** 10.1186/s12911-023-02311-3

**Published:** 2023-10-09

**Authors:** April R. Williams, Maria D. Thomson, Erin L. Britton

**Affiliations:** Health Behavior and Policy, Virginia Commonwealth University, 830 E Main Street, Richmond, VA 23219 USA

**Keywords:** Chronic disease, Hypertension, Preventive care, Electronic health records, Nutrition

## Abstract

**Background:**

Clinical events suggestive of nutrition care found in electronic health records (EHRs) are rarely explored for their associations with hypertension outcomes.

**Methods:**

Longitudinal analysis using structured EHR data from primary care visits at a health system in the US from December 2017—December 2020 of adult patients with hypertension (*n* = 4,237) tested for associations between last visit blood pressure (BP) control (≤ 140 Systolic BP and ≤ 90 Diastolic BP) and ≥ 1 nutrition care clinical event operationalized as (overweight or obesity (BMI > 25 or 30, respectively) diagnoses, preventive care visits, or provision of patient education materials (PEM)). Descriptive statistics and longitudinal targeted maximum likelihood estimation (LTMLE) models were conducted to explore average treatment effects (ATE) of timing and dose response from these clinical events on blood pressure control overall and by race.

**Results:**

The median age was 62 years, 29% were male, 52% were Black, 25% were from rural areas and 50% had controlled BP at baseline. Annual documentation of overweight/obesity diagnoses ranged 3.0–7.8%, preventive care visits ranged 6.2–15.7%, and PEM with dietary and hypertension content were distributed to 8.5–28.8% patients. LTMLE models stratified by race showed differences in timing, dose, and type of nutrition care. Black patients who had nutrition care in Year 3 only compared to none had lower odds for BP control (ATE -0.23, 95% CI: -0.38,-0.08, *p* = 0.003), preventive visits in the last 2 years high higher odds for BP control (ATE 0.31, 95% CI: 0.07,0.54, *p* = 0.01), and early or late PEMs had lower odds for BP control (ATE -0.08, 95% CI: -0.15,-0.01, *p* = 0.03 and ATE -0.23, 95% CI: -0.41,-0.05, *p* = 0.01, respectively).

**Conclusions:**

In this study, clinical events suggestive of nutrition care are significantly associated with BP control, but are infrequent and effects differ by type, timing, and patient race. Preventive visits appear to have the most effect; additional research should include examining clinical notes for evidence of nutrition care among different populations, which may uncover areas for improving nutrition care for patients with chronic disease.

**Supplementary Information:**

The online version contains supplementary material available at 10.1186/s12911-023-02311-3.

## Background

High blood pressure (BP), or hypertension, affects nearly 1 in 3 US adults [1], with approximately half of cases under poor control, increasing the risk of death from a sudden heart attack or stroke [2]. A variety of clinical events suggestive of nutrition care that occur in primary care settings may have a positive effect on patients’ dietary patterns, and therefore promote blood pressure control [3]. These could be in the form of the awareness brought to the patient through an obesity or overweight diagnosis [4], providers or staff spending time counseling a patient about the importance of diet to prevent, treat, or manage a chronic disease during preventive care visits [5, 6], or patient education materials (PEMs) containing nutrition information relevant to chronic disease management provided in an after-visit summary [7].

Providing nutrition care may improve patient self-management and hypertension outcomes overall [8, 9], but many primary care clinicians and staff face substantial time barriers to conducting and documenting these activities in electronic health records [10, 11]. Rates of nutrition care events are generally low [5, 12], and their documentation in structured EHR data is largely unknown [13, 14]. A lack of documentation in the EHR may result in limited communication within the care team about patients’ nutrition statuses and challenges in evaluating care quality, which may be a missed opportunity to improve patient outcomes [15].

Little is known about the rates of nutrition care events documented for hypertension management or their relationship with BP control of patients. Given this, the objectives of this study were to assess of rates of nutrition care events identified in structured EHR data and to test whether the timing and dose of these were associated with BP control for adult patients in an academic health system. The hypothesis tested was that documented nutrition care, controlling for patient demographic and health characteristics, is associated with BP control.

## Methods

### Study design and setting

A single, safety-net academic medical center with six primary care clinic sites in the Mid-Atlantic US serves a diverse patient population of both rural and urban, and underrepresented patients. Deidentified EHR data were obtained for clinical visits (*n* = 43,246) between December 2017 and December 2020 for (*n*= 4,237) adult patients, age 18 to 85 years at the date of their last BP measurement, who had their first visit prior to 1/1/2019 with a recorded BP measurement, at least two primary care visits during the study period and had a previous diagnosis of hypertension. Records of patients with chronic kidney disease, pregnancy, or who were in hospice or long-term care were excluded per the clinical quality measure for controlling high BP [16]. Patient data included demographics: age, sex, racial/ethnicity categories, rural geography indicated by Rural Urban Continuum codes 4–9 [17], and insured status; and clinical factors relevant to hypertension: BMI, comorbidities [18], hypertension medications (see Supplemental Materials) [19], number of visits, and indicators for clinical events suggestive of nutrition care and BP control [16].

### Blood pressure control

The outcome of interest was BP control (yes/no), defined as ≤ 140 mm Hg systolic BP (SBP) and ≤ 90 mm Hg diastolic BP (DBP) per the National Committee for Quality Assurance (NCQA) quality measure for “Controlling High Blood Pressure”. This outcome was calculated at the patients’ first visit and annually based on the last measured BP in the calendar year. BP control values were carried forward if they were not updated in subsequent visits.

### Operationalizing nutrition care

Three commonly provided services in primary care hypertension management were used as a proxy for nutrition care, justified by the assumption that nutrition-related information was exchanged with the patient. These nutrition care events were exclusive of the final visit and identified by diagnosis and billing codes for overweight/obesity diagnoses and preventive visits, and a flag that diet-related education materials were provided to the patient within the study period. Clinical guidelines for the treatment of overweight/obesity include dietary behavior management [20, 21], and the diagnosis itself may inherently offer an opportunity as a “teachable moment” behavior change intervention [4]. Preventive visits that include evaluation for and management of chronic diseases like hypertension were chosen for the comprehensive counseling and guidance provided to patients to reduce risk factors that include diet [22, 23]. Providing patients with printed education materials is a common and passive method for counseling patients about dietary recommendations.

### Analyses

Descriptive statistics (frequencies, proportions, medians, and ranges) were calculated for all patients’ and stratified samples of Black and white patients’ demographic characteristics and hypertension-relevant indicators, proportions of patients with controlled BP, and rates of documented nutrition care events.

**Fig. 1:**
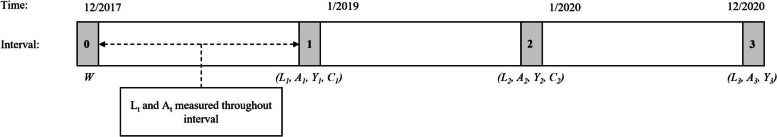
Temporal order of EHR visit data coarsened into yearly intervals. The baseline variables (W) included race (African American or Black/White), rural, sex, age^2^, # comorbidities were recorded at the first visit of the study window. Each follow-up interval included time-varying covariates (L,; # hypertension medications and # primary care visits), an indicator of having received any nutrition care events during the interval (A_t_); and an indicator of blood pressure control (Y_t_) recorded at the last visit of each calendar year. The censoring variable (C_t_) indicates a patient did not return for follow-up

Longitudinal Targeted Maximum Likelihood Estimate (LTMLE) [24, 25] is a doubly-robust, semiparametric approach that combines sequential g-computation and inverse probability weighting to estimate an effect of a longitudinal treatment (in this case, clinical events suggestive of nutrition care) respecting its temporal relationship with an outcome (BP control). The LTMLE package [26] for R was used to estimate average treatment effects (ATE) of nutrition care events (i. any nutrition care event; ii. overweight or obesity diagnoses; iii. preventive care visits; and iv. provision of patient education materials) on patients’ BP control (yes/no) over the study period and stratified among data for Black patients and white patients. See Fig. 1 for an explanation of the data shaping process used for the models. The models adjusted for fixed covariates: age, sex, race/ethnicity, geography (rural/urban), comorbidities; and time-varying covariates: number of hypertension medications prescribed and number of primary care visits annually during the study period. The models were stratified by race as a way to separately examine [27] the strength of association between controlled BP and nutrition care events among Black patient and white patient groups. Sensitivity analyses were conducted with insured status and BMI, as well as elimination of Year 3 (2020) due to the substantial reduction in recorded primary care visits due to the COVID-19 pandemic.

Results are presented as average treatment effect parameter estimate differences (ATE), 95% confidence intervals, and *p*-values for each LTMLE model with alpha 0.05. Comorbidities were calculated with HCUP Elixhauser software [18] program using SAS Enterprise Edition 3.7 (SAS Institute, Cary, North Carolina, USA). All remaining analyses were conducted using R Statistical Software (Version 4.0.3; R Foundation for Statistical Computing, 2020). This study was approved as exempt by the Institutional Review Board at Virginia Commonwealth University.

## Results

Descriptive statistics for patients’ characteristics and clinical factors are found in Table 1. The overall median age of patients in the EHR data sample (*n* = 4,237) was 62 years (range:18,85), nearly a third (*n* = 1,217; 28.7%) were male, half (*n* = 2,198; 51.9%) were Black/African American, 25.4% (*n* = 1,077) were from rural areas, and 7.9% (*n* = 333) were covered by Medicaid insurance. Most patients in the sample were overweight or obese (*n* = 3,843; 90.7%) for at least one recorded BMI during the study period. The median total number of anti-hypertensive prescriptions per patient over the study period was 6 (range:0,50). About a third (*n* = 1,550; 36.6%) had one or more comorbidities, and less than half (*n* = 2,103; 49.6%) had controlled BP at their first recorded BP during the study period.

**Table 1:** Patient demographics and hypertension risk factors

	**Total Patients**^**a**^** (*n* = 4,237)**	**Black Patients(*n* = 2,198)**	**White Patients(*n* = 2,037)**
**Median Age (range)**	62 (18,85)	59 (18,85)	66 (18,85)
**Male n (%)**	1,215 (28.7)	487 (22.2)	728 (35.7)
**Race**^**b**^** n (%)**			
African American or Black	2,198 (51.9)	-	-
White	2,039 (48.1)	-	-
**Rural**^**c**^** n (%)**	1,077 (25.4)	314 (14.3)	763 (37.4)
**Medicaid n (%)**	333 (7.9)	256 (11.6)	77 (53.8)
**Overweight or Obese BMI**^**d**^** (%)**	3,843 (90.7)	2,037 (92.7)	1,806 (88.5)
**Median Hypertension Medications**^**e**^** (range)**	6 (0,50)	7 (0,50)	6 (0,31)
**One or More Comorbidities**^**f**^** n (%)**	1,550 (36.6)	896 (40.8)	652 (32.1)
**Median Number of Visits (range)**	9 (0,91)	9 (0,84)	9 (0,91)
**Controlled Blood Pressure**^**g**^** n (%)**			
**Baseline**	2,103 (49.6)	1,051 (47.8)	1,052 (51.6)
**Year 1**	2,437 (57.5)	1,230 (56.0)	1,207 (59.2)
**Year 2**	2,345 (55.4)	1,172 (53.3)	1,173 (57.5)
**Year 3**	1,920 (45.3)	943 (42.9)	977 (47.9)

^a^Number of patients based on patient’s last visit

^b^Includes both Hispanic and non-Hispanic African American/Black and Whites

^c^Patients residing in rural areas was determined using patient’s zip code corresponding to RUCC code (4–9)

^d^Overweight or obese categories derived from BMI is calculated as the maximum recorded BMI across the study period per patient If BMI was not available, the patient’s height and maximum weight across the study period were used to calculate BMI using the standard formula (weight (kg) / height (cm) / height (cm)] × 10,000)

^e^Hypertension medications were the total prescribed across the study period per patient. Medications were identified using the HEDIS reference list for hypertension medications [28] and reviewed with two physicians boarded in internal medicine prior to making calculations

^f^Comorbidities calculated as mode of Elixhauser Comorbidity Index across the study period per patient. The index was calculated using the Healthcare Cost and Utilization Project Methods Series Comorbidity Software. The comorbidity index is a total sum of diagnosed chronic disease comorbidities found in 31 categories defined in the software [21].

^g^Patients with controlled hypertension (≤ 140 mm Hg SBP and ≤ 90 mm Hg DBP) per the National Committee for Quality Assurance (NCQA) quality measure for “Controlling High Blood Pressure” [22] were flagged (yes/no). BP was calculated at the patients’ first visit and annually based on the last measured BP in the calendar year

Table 2 shows a summary of documented nutrition care event rates by year among all the patients in the sample, as well as rates stratified by Black and white patient groups. The median number of nutrition care events documented in the EHR data was 1(range:0,16) per patient during the study period, with substantially fewer in Year 3 (2020). Distribution of PEMs was the most frequent nutrition care event documented each year (range overall: *n* = 533 (16.4%) to *n* = 1,855 (43.8%); for Black patients: *n* = 125 (7.6%) to *n* = 617 (28.1%); and for white patients: *n* = 153 (9.5%) to *n* = 602 (29.5%)). Overweight/obesity diagnoses were recorded for far fewer patients annually across the study period (range overall: *n* = 98 (3.0%) to *n* = 329 (7.8%); for Black patients: *n* = 61 (3.7%) to *n* = 210 (10.3%); and for white patients: *n* = 37 (2.3%) to *n* = 125 (6.1%)). Ranges of documented preventive visits were overall: *n* = 203 (6.2%) to *n* = 664 (15.7%); for Black patients: *n* = 85 (5.1%) to *n* = 319 (14.5%); and white patients: *n* = 118 (7.4%) to *n* = 345 (16.9%).

**Table 2:** Rates of nutrition care events

	**Total Patients** **(*n* = 4,237)**	**Black Patients** **(*n* = 2,198)**	**White Patients** **(*n* = 2,039)**
Median Nutrition Care^a^ (range)	1 (0, 16)	1 (0, 16)	1 (0, 15)
Nutrition Care^b^ n (%)			
Year 1	1,855 (43.8%)	950 (43.2%)	905 (44.4%)
Year 2	1,538 (39.0%)	831 (40.6%)	707 (37.3%)
Year 3	533 (16.4%)	251 (15.2%)	282 (17.6%)
Preventive Visits^e^ n (%)			
Year 1	664 (15.7%)	319 (14.5%)	345 (16.9%)
Year 2	573 (14.5%)	268 (13.1%)	305 (16.1%)
Year 3	203 (6.2%)	85 (5.1%)	118 (7.4%)
Overweight/Obesity Diagnosis^d^ n (%)			
Year 1	329 (7.8%)	204 (9.3%)	125 (6.1%)
Year 2	295 (7.5%)	210 (10.3%)	85 (4.5%)
Year 3	98 (3.0%)	61 (3.7%)	37 (2.3%)
Patient Education Materials^c^ n (%)			
Year 1	1,219 (28.8%)	617 (28.1%)	602 (29.5%)
Year 2	965 (24.5%)	530 (25.9%)	435 (23.0%)
Year 3	278 (8.5%)	125 (7.6%)	153 (9.5%)
Missing Blood Pressure^f^			
Year 2	296 (7.0%)	151 (6.9%)	145 (7.1%)
Year 3	983 (23.2%)	547 (24.9%)	436 (21.4%)

^a^Number of nutrition care services documented per patient across the study period. Services were flagged (yes/no) based on the list of International Classification of Diseases version 10 (ICD-10) diagnosis and Current Procedural Terminology (CPT) codes

^b^Number of patients who received at least one form of nutrition care across the study period

^c^Number of patients who received at least one patient education material (PEM) across the study period

^d^Number of patients who were diagnosed with overweight or obesity (ICD-10: Z68.XX; Z71.3; R63.5; E66.XX; E88.81) across the study period

^e^Number of patients who were provided with a preventive care visit (CPT: 99,381–99,387, 99,391–99,397) that includes counseling about chronic disease risk factors and management strategies across the study period

^f^Proportions of nutrition care services for years 2 and 3 are based on patients who were not censored: Year 1: *n* = 3,941; Year 2: *n* = 3,254

### Timing of and repeated nutrition care events effects on BP control

Estimates of average treatment effects (ATE) on BP control for any nutrition care event, at least one of preventive care visits, obesity/overweight diagnosis, or provision of PEMs at different timing intervals – Year 1 (Early), Year 3 (Late) – compared to none of these clinical visits are presented in Table 3. Any nutrition care events recorded in year 3, compared with none, were inversely associated with BP control for the overall sample and for Black patients (ATE -0.12, 95% CI: -0.24,-0.01, *p* = 0.03 and ATE -0.23, 95% CI: -0.38,-0.08, *p* = 0.003, respectively). Having a nutrition care event in both years 1 and 3 compared to none was positively associated with BP control (ATE 0.16, 95% CI: 0.00,0.32, *p* = 0.046) among Black patients, however.

**Table 3:** Estimated average treatment effects of nutrition care events^a^ on blood pressure control

	**Blood Pressure ControlRisk Difference (RD) (95% CI; *p*-value)**			
	**Any Nutrition Care Events**	**Preventive Care Visits**	**Overweight/Obesity Diagnoses**	**Patient Education Materials**
**Estimates vs. E(Y(0,0,0))**^**b**^				
**Early E(Y(1,0,0))**	-0.05, (-0.10,0.01; 0.09)	0.04, (-0.03,0.11; 0.264)	-0.01, (-0.10,0.08; 0.83)	**-0.06, (-0.11,-0.01; 0.016)**
**Late E(Y(0,0,1))**	**-0.12, (-0.24,-0.01; 0.03)**	0.12, (-0.03,0.26; 0.11)	0.01, (-0.14,0.15; 0.92)	**-0.15, (-0.28,-0.02; 0.02)**
**Early + Late E(Y(1,0,1))**	0.08, (-0.04,0.20; 0.19)	-0.08, (-0.23,0.08; 0.35)	-0.02, (-0.19,0.15; 0.84)	0.09, (-0.04,0.22; 0.185)
**All E(Y(1,1,1))**	0.00, (-0.08,0.09; 0.93)	-0.05, (-0.22,0.11; 0.53)	0.09, (-0.28,0.45; 0.64)	0.01, (-0.12,0.15; 0.826)
**More Early E(Y(1,1,0))**	-0.02, (-0.07,0.03; 0.40)	0.06, (-0.04,0.16; 0.22)	0.09, (-0.06,0.25; 0.25)	-0.01, (-0.08,0.05; 0.684)
**More Late E(Y(0,1,1))**	-0.03, (-0.16,0.09; 0.60)	0.15, (-0.06,0.36; 0.15)	-0.1, (-0.65,0.45; 0.73)	-0.15, (-0.35,0.04; 0.118)
**Estimates vs. E(Y(0,0,0)) among Black Patients**				
**Early E(Y(1,0,0))**	-0.07, (-0.14,0.01; 0.09)	0.08, (-0.02,0.18; 0.10)	0, (-0.13,0.13; 0.99)	**-0.08, (-0.15,-0.01; 0.03)**
**Late E(Y(0,0,1))**	**-0.23, (-0.38,-0.08; 0.003)**	0.15, (-0.06,0.35; 0.15)	-0.05, (-0.24,0.13; 0.58)	**-0.23, (-0.41,-0.05; 0.01)**
**Early + Late E(Y(1,0,1))**	**0.16, (0.00,0.32; 0.046)**	-0.07, (-0.29,0.16; 0.56)	0.05, (-0.17,0.27; 0.64)	0.15, (-0.03,0.34; 0.11)
**All E(Y(1,1,1))**	-0.05, (-0.17,0.07; 0.45)	-0.02, (-0.30,0.26; 0.89)	-0.09, (-0.61,0.44; 0.75)	-0.04, (-0.22,0.15; 0.70)
**More Early E(Y(1,1,0))**	-0.06, (-0.14,0.02; 0.13)	0.09, (-0.06,0.25; 0.25)	0.06, (-0.13,0.24; 0.56)	-0.03, (-0.13,0.06; 0.51)
**More Late E(Y(0,1,1))**	0.06, (-0.11,0.22; 0.48)	**0.31, (0.07,0.54; 0.01)**	-0.16, (-0.66,0.34; 0.52)	-0.15, (-0.42,0.12; 0.28)
**Estimates vs. E(Y(0,0,0)) among white Patients**				
**Early E(Y(1,0,0))**	-0.04, (-0.12,0.04; 0.35)	-0.03, (-0.13,0.07; 0.55)	-0.05, (-0.19,0.10; 0.51)	-0.04, (-0.11,0.03; 0.30)
**Late E(Y(0,0,1))**	0.01, (-0.15,0.17; 0.87)	**0.19, (0.01,0.38; 0.04)**	0.12, (-0.15,0.39; 0.38)	-0.07, (-0.25,0.11; 0.46)
**Early + Late E(Y(1,0,1))**	-0.01, (-0.18,0.17; 0.95)	-0.03, (-0.38,0.31; 0.85)	-0.18, (-0.86,0.49; 0.59)	0.00, (-0.20,0.21; 0.98)
**All E(Y(1,1,1))**	0.05, (-0.10,0.20; 0.50)	-0.06, (-0.28,0.16; 0.57)	0.21, (-0.26,0.68; 0.37)	0.07, (-0.11,0.26; 0.45)
**More Early E(Y(1,1,0))**	0.00, (-0.08,0.08; 0.99)	0.03, (-0.11,0.17; 0.70)	0.23, (-0.02,0.48; 0.07)	0.00, (-0.10,0.09; 0.95)
**More Late E(Y(0,1,1))**	-0.15, (-0.34,0.03; 0.11)	0.00, (-0.34,0.34; 0.99)	**0.41, (0.38,0.43; < 0.001)**	-0.23, (-0.51,0.04; 0.10)

^a^Longitudinal Targeted Maximum Likelihood Estimate (LTMLE) model: Fixed covariates: Race (African American or Black / White), Rural, Sex, Age^2^, # Comorbidities; Time-varying covariates: # hypertension medications at baseline, end Year 1, end Year 2, end Year 3; # primary care visits end Year 1, end Year 2, end Year 3; Independent Variable: >  = 1 Nutrition Care Events at end Year 1, end Year 2, end Year 3; Outcome Variable: Blood Pressure Control (yes/no); Censoring indicator for missing outcome at end Year 2 and end Year 3

^b^Notation example: E(Y(1,0,0)) vs. E(Y(0,0,0)): Expected risk difference for at least one nutrition care event during Year 1, but none in Years 2 or 3, compared to none across the study period

Preventive visits in years 2 and 3 versus none among Black patients had a positive effect on BP control (ATE 0.31, 95% CI: 0.07,0.54, *p* = 0.01). Similarly, preventive visits in only year 3 versus non among white patients had a positive effect on BP control (ATE 0.19, 95% CI: 0.01,0.38, *p* = 0.04). Obesity/overweight diagnoses during both years 2 and 3 versus none among white patients had the greatest effect on BP control in the study (ATE 0.41, 95% CI: 0.38,0.43, *p* < 0.001). Patient Education materials early (year 1) or late (year 3) versus none both had negative and significant associations with BP control for the full sample (ATE -0.06, 95% CI: -0.11,-0.01, *p* = 0.016 and ATE -0.15, 95% CI: -0.28,-0.02, *p* = 0.02, respectively). This was also true among Black patients (ATE -0.08, 95% CI: -0.15,-0.01, *p* = 0.03 and ATE -0.23, 95% CI: -0.41,-0.05, *p* = 0.01, respectively).

## Discussion

EHR data was used in this study to examine associations between BP control and nutrition care events identified through documentation of clinical activities that imply nutrition or diet information was communicated with patients who have hypertension. Visits reported in the EHR data were at primary care clinics part of a health system that serves a diverse population of rural and underrepresented racial/ethnic patients. Among the 4,237 patients with hypertension, rates of nutrition care events were generally low, although number of documented nutrition care events, preventive care visits where guidelines suggest counseling about dietary risk factors take place were found to be associated with improved odds for BP control. However, there were disparities in BP control and rates of clinical events suggestive of nutrition care recorded by race.

Overall low rates of documented nutrition care events found in this study support the wider call to address barriers faced by clinicians and staff such as time pressures and limited provider education in providing nutrition care in primary care settings [10, 11]. To understand how these rates might increase, these clinical activities must be measured, and to be measured, they must be documented. Stange and colleagues argued health systems measure what is valued [29], and this study highlights a few important opportunities where nutrition is addressed with patients: timing and frequency (dose) of diagnoses of overweight or obesity diagnoses, preventive care visits, and provision of patient education materials.

In this study, an overweight or obesity diagnosis was only associated with controlled BP among white patients when recorded in both years 2 and 3 compared with no record or diagnosis. However, BMI persists as a clinical tool for risk assessment, and the diagnosis, as previously mentioned, could play an important role in patient awareness of a health risk. Mixed findings about BMI associations with hypertension management is seen in a recent study of BP control rates among adults with hypertension. Foti and colleagues found those with overweight or obesity had higher rates of BP control compared with those who had lower BMI. This, the authors suggested, might be because the lower BMI patients may be less aware of their hypertension and be treated less often or intensely [30]. Interestingly, rates of obesity and overweight diagnoses were not concordant with BMI calculated from patient chart data in the present study. The disconnect between BMI and documented obesity diagnoses has been shown elsewhere; one study of EHR data found that while more than half (52%) of patients in the sample had a BMI ≥ 30.0 qualifying them for an obesity diagnosis, very few (5.6%) had obesity recorded in their health record problem list [31]. One factor contributing to the lack of documenting or addressing overweight or obesity with patients may be clinicians are ill-prepared to discuss the topic [32].

Few patients in the present study had at least one preventive visit that included a discussion of nutrition-related risk factors documented in their EHR record. Clinical guidelines suggest nutrition counseling is included in lifestyle treatment for chronic diseases for which diet is a risk factor. Healthy People 2020 suggested a goal to “*increase the proportion of physician office visits made by patients with a diagnosis of cardiovascular disease, diabetes, or hyperlipidemia that include counseling or education related to diet or nutrition,*” [33] from 11.5% to 12.7% (a 10% increase) by 2020 as measured by the National Ambulatory Medical Care Survey (NAMCS). Overall rates reached over 20% by 2015, which may be why the objective was eliminated altogether for Healthy People 2030. However, rates assessed by the present study and Healthy People remain objectively low for a service that may be universally useful if it were achievable to provide such service to all patients, as preventive visits have long been shown to help improve patient health outcomes.

Of note, preventive care visits were less common for Black patients compared to white patients. Given BP control was less likely among Black patients, yet preventive care visits in years 2 and 3 appeared to have the strongest positive and significant effect on BP control compared to no preventive care visits. This suggests a disparity in service uptake that may be a missed opportunity for which further understanding and improvements are needed. Others have researched and found delays and underutilization of health services by Black patients due to a variety of reasons [34] including socioeconomic barriers [35], medical mistrust [36] and perceived racism [37].

Leveraging data entered into the patient’s EHR is a way to support the care team in prioritizing appropriate preventive care depending upon patients’ individual needs. Krist, et. al. developed an EHR intervention that incorporated automated, tailored, patient-centered messaging for preventive care. In a summary of the initial six months of its use (November 2010 through May 2011), clinics were successful in using the tool to support clinicians to counsel patients about health behaviors and customize prevention and treatment plans for patients [38]. Such initiatives have been instituted within various health systems and clinics nationally over the past decade to support clinicians through the use of risk calculators [39], automated prompts for clinicians and patients [40], and provision of printed or electronic patient education materials [41]. Although patient education materials were inversely and significantly associated with BP control in this study, they were the most commonly provided nutrition care event. Timely research is needed to understand the effects of communicating nutrition-related chronic disease prevention and management in primary care. Due to the fast rate at which technology use in health care has advanced, health services researchers face additional challenges in efficient dissemination and evaluation of EHR process implementations [42].

Findings of this study offer new contributions and suggestions for additional work needed within the burgeoning area of health services research that seeks to improve preventive services to support patients with chronic disease. First, due to our limited established structures to measure or examine nutrition-related activities in primary care, this study provides a framework of proxy measures for preventive nutrition care that may be found in the voluminous EHR data. Next, this study applied longitudinal analyses in stratified samples of underrepresented racial and ethnic patients, with significant findings to support conducting further detailed explorations of causes and ways forward for improvement. Although not part of the present study, additional stratification by comorbidities may be conducted to assess differences in treatment plans and nutrition care for complex patients. To gain more insight into nutrition care services and relevant patient outcomes, future research should use a larger data sample or data from a group of health systems, and additional data sources e.g., clinical notes for preventive counseling; and claims data for clinical referrals to dietitians are other avenues to offer a more robust picture of successful processes for conducting and documenting nutrition care delivery that might reveal targets for improvements.

Our study has important limitations. Using EHR data for research has known challenges due to variability of data entry [43]. As a single health system study, findings may not be generalizable, clinics part of the health system served diverse patient populations that represent a higher risk for health disparities. Measurement bias needs to be considered in the study findings, given there may have been some proportion of clinical visits in virtual format at the onset of COVID. We attempted to correct for as much bias as possible with inclusion criteria described in the Methods and conducted a sensitivity analysis in models that excluded year 3 (2020) and found similar general results. Furthermore, the proxy measures for nutrition care used in this study were not manually confirmed with chart review and would be an interesting future study. These services are critical to chronic disease management and challenges related to their documentation and delivery to patients remain globally applicable.

## Conclusions

The present study utilized EHR data to provide a description of nutrition care delivery and examination of its association with patient BP outcomes within a single health system. Overall, documentation of received nutrition care events was low, and preventive care visits, but not overweight/obesity diagnosis or delivery of patient education materials, was associated with patients’ BP control. Additionally, disparities were identified in rates of nutrition care events and odds for BP control by race. Further research is needed to explore ways to improve documentation and equity of nutrition care. Enhancing EHR workflows, education for providers and staff about nutrition care, and improving effective communication and collaboration within the clinical team may all be system-level targets for improving nutrition care and hypertension outcomes.

### Supplementary Information

**Additional file 1.**

## Data Availability

The datasets generated and/or analyzed during the current study are not publicly available due to the data use agreement from source health records. Source code and analytic files are available from the corresponding author on reasonable request.

## Abbreviations

HER: Electronic health records
BP: Blood pressure
PEM: Patient education materials
BMI: Body mass index
SBP: Systolic blood pressure
DBP: Diastolic blood pressure
NCQA: National Committee for Quality Assurance
LTMLE: Longitudinal Targeted Maximum Likelihood Estimate
ATE: Average treatment effects
COVID-19: Corona Virus Disease 2019
HCUP: Healthcare Cost and Utilization Project
SAS: Statistical Analysis Software

## References

[1] Ostchega Y, Fryar CD, Nwankwo T, Nguyen DT (2020). Hypertension prevalence among adults aged 18 and over: United States, 2017–2018. NCHS Data Brief.

[2] Whelton PK, Carey RM, Aronow WS (2018). 2017 ACC/AHA/AAPA/ABC/ACPM/AGS/APhA/ ASH/ASPC/NMA/PCNA guideline for the prevention, detection, evaluation, and management of high blood pressure in adults a report of the American College of Cardiology/American Heart Association Task Force on Clinical pr. Hypertension.

[3] Lacey K, Pritchett E (2003). Nutrition care process and model: ADA adopts road map to quality care and outcomes management. J Am Diet Assoc.

[4] Coa KI, Smith KC, Klassen AC (2015). Capitalizing on the “teachable moment” to promote healthy dietary changes among cancer survivors: the perspectives of health care providers. Support Care Cancer.

[5] Grabovac I, Smith L, Stefanac S (2019). Health Care Providers’ Advice on Lifestyle Modification in the US Population: Results from the NHANES 2011–2016. Am J Med.

[6] Williams AR, Wilson-Genderson M, Thomson MD (2021). A cross-sectional analysis of associations between lifestyle advice and behavior changes in patients with hypertension or diabetes: NHANES 2015–2018. Prev Med.

[7] Lyles CR, Gupta R, Tieu L, Fernandez A (2019). After-visit summaries in primary care: mixed methods results from a literature review and stakeholder interviews. Fam Pract.

[8] Kravetz JD, Walsh RF (2016). Team-based hypertension management to improve blood pressure control. J Prim Care Community Health.

[9] Kaboli PJ, Howren MB, Ishani A, Carter B, Christensen AJ, Vander Weg MW (2018). Efficacy of Patient Activation Interventions With or Without Financial Incentives to Promote Prescribing of Thiazides and Hypertension Control. JAMA Netw Open.

[10] Kushner RFF (1995). Barriers to Providing Nutrition Counseling by Physicians: A Survey of Primary Care Practitioners. Prev Med.

[11] Bardach SH, Schoenberg NE (2012). Primary care physicians’ prevention counseling with patients with multiple morbidity. Qual Health Res.

[12] Aspry KE, Linda Van Horn C, Chair Jo Ann Carson VS, et al. Medical Nutrition Education, Training, and Competencies to Advance Guideline-Based Diet Counseling by Physicians. Circulation. 2018;137(1):821–41. 10.1161/CIR.0000000000000563 10.1161/CIR.0000000000000563 29712711

[13] Hosomura N, Goldberg SI, Shubina M, Zhang M, Turchin A (2015). Electronic documentation of lifestyle counseling and glycemic control in patients with diabetes. Diabetes Care.

[14] Bae J, Hockenberry JM, Rask KJ, Becker ER (2017). Evidence that electronic health records can promote physician counseling for healthy behaviors. Health Care Manage Rev.

[15] Watterson JL, Rodriguez HP, Aguilera A, Shortell SM (2020). Ease of use of electronic health records and relational coordination among primary care team members. Health Care Manage Rev.

[16] CQF. 2019 HEDIS ® Measures Controlling High Blood Pressure (CBP) HEDIS Measure Description. www.ncqa.org. Accessed 21 Dec 2019.

[17] USDA ERS - Rural-Urban Continuum Codes. https://www.ers.usda.gov/data-products/rural-urban-continuum-codes.aspx. Accessed 23 Oct 2019.

[18] HCUP Methods Series Comorbidity Software Documentation. https://www.hcup-us.ahrq.gov/reports/methods/2004_1.jsp.

[19] HEDIS MY 2020 Medication List Directory. https://store.ncqa.org/hedis-my-2020-medication-list-directory.html. Accessed 18 June 2021.

[20] Obesity in adults: Overview of management - UpToDate. https://www-uptodate-com.proxy.library.vcu.edu/contents/obesity-in-adults-overview-of-management?search=obesity%20management&source=search_result&selectedTitle=1~150&usage_type=default&display_rank=1. Accessed 16 July 2022.

[21] Curry SJ, Krist AH, US Preventive Services Task Force (2018). Behavioral weight loss interventions to prevent obesity-related morbidity and mortality in adults: US Preventive services task force recommendation Statement. JAMA..

[22] Diet in the treatment and prevention of hypertension - UpToDate. https://www.uptodate.com/contents/diet-in-the-treatment-and-prevention-of-hypertension. Accessed 16 Aug 2019.

[23] Casey DE, Thomas RJ, Bhalla V (2019). 2019 AHA/ACC clinical performance and quality measures for adults with high blood pressure: a report of the American college of cardiology/American heart association task force on performance measures. Circ Cardiovasc Qual Outcomes.

[24] Petersen M, Schwab J, Gruber S, Blaser N, Schomaker M, van der Laan M (2014). Targeted maximum likelihood estimation for dynamic and static longitudinal marginal structural working models. J Causal Inference.

[25] Schnitzer ME, van der Laan MJ, Moodie EEM, Platt RW. LTMLE with Clustering. In: van der Laan MJ, Rose S, eds. Targeted Learning in Data Science: Causal Inference for Complex Longitudinal Studies. Springer Series in Statistics. Springer International Publishing; 2018:233–251. 10.1007/978-3-319-65304-4_15.

[26] Lendle SD, Schwab J, Petersen ML, van der Laan MJ (2017). ltmle: an R package implementing targeted minimum loss-based estimation for longitudinal data. J Stat Softw.

[27] Ward JB, Gartner DR, Keyes KM, Fliss MD, McClure ES, Robinson WR (2019). How do we assess a racial disparity in health? Distribution, interaction, and interpretation in epidemiological studies. Ann Epidemiol.

[28] HEDIS MY 2020 Medication List Directory. https://store.ncqa.org/hedis-my-2020-medication-list-directory.html. Accessed 18 June 2021

[29] Stange KC, Etz RS, Gullett H (2014). Metrics for assessing improvements in primary health care. Annu Rev Public Health.

[30] Foti K, Hardy ST, Chang AR, Selvin E, Coresh J, Muntner P (2022). BMI and blood pressure control among United States adults with hypertension. J Hypertens.

[31] Mattar A, Carlston D, Sariol G (2017). The prevalence of obesity documentation in primary care electronic medical records: Are we acknowledging the problem?. Appl Clin Inform.

[32] Antognoli EL, Seeholzer EL, Gullett H, Jackson B, Smith S, Flocke SA (2017). Primary Care Resident Training for Obesity, Nutrition, and Physical Activity Counseling: A Mixed-Methods Study. Health Promot Pract.

[33] Nutrition and Weight Status | Healthy People 2020. https://www.healthypeople.gov/2020/topics-objectives/topic/nutrition-and-weight-status. https://www.healthypeople.gov/2020/topics-objectives/topic/nutrition-and-weight-status. Accessed 16 Mar 2020.

[34] Powell W, Richmond J, Mohottige D, Yen I, Joslyn A, Corbie-Smith G (2019). Medical mistrust, racism, and delays in preventive health screening among African-American men. Behav Med Wash DC.

[35] Ravenell JE, Whitaker EE, Johnson WE (2008). According to him: barriers to healthcare among African-American men. J Natl Med Assoc.

[36] Hammond WP, Matthews D, Mohottige D, Agyemang A, Corbie-Smith G (2010). Masculinity, medical mistrust, and preventive health services delays among community-dwelling African-American Men. J Gen Intern Med.

[37] Cheatham CT, Barksdale DJ, Rodgers SG (2008). Barriers to health care and health-seeking behaviors faced by Black men. J Am Acad Nurse Pract.

[38] Krist AH, Peele E, Woolf SH (2011). Designing a patient-centered personal health record to promote preventive care. BMC Med Inform Decis Mak.

[39] McGuinness JE, Zhang TM, Cooper K (2022). Extraction of Electronic Health Record Data using Fast Healthcare Interoperability Resources for Automated Breast Cancer Risk Assessment. AMIA Annu Symp Proc.

[40] Krist AH (2015). Electronic Health Record Innovations for Healthier Patients and Happier Doctors. J Am Board Fam Med JABFM.

[41] Schooley B, Singh A, Hikmet N, Brookshire R, Patel N (2020). Integrated digital patient education at the bedside for patients with chronic conditions: observational study. JMIR MHealth UHealth.

[42] Brownson RC, Eyler AA, Harris JK, Moore JB, Tabak RG (2018). Getting the word out: new approaches for disseminating public health science. J Public Health Manag Pract.

[43] Casey JA, Schwartz BS, Stewart WF, Adler NE (2016). Using electronic health records for population health research: a review of methods and applications HHS public access. Annu Rev Public Health.

